# Retrospective study of patellar fractures and damage of accessory soft tissue based on MRI

**DOI:** 10.1371/journal.pone.0295671

**Published:** 2024-03-11

**Authors:** Yi-Fan Hua, Yu-Rou Zhang, Li Guo

**Affiliations:** 1 Department of Radiology, Second Affiliated Hospital of Kunming Medical University, Kunming, Yunnan, P.R. China; 2 Department of School Infirmary, Yunnan Minzu University, Kunming, Yunnan, P.R. China; Sheikh Hasina National Institute of Burn & Plastic Surgery, BANGLADESH

## Abstract

**Background:**

To better understand the pathophysiological mechanisms of patellar fractures, MRI was utilized to identify the imaging signs of various types of patellar fractures.

**Methods:**

A retrospective study was performed using MRI images of 52 patients with patellar fractures. Observing the development of patellar and femoral morphology and the imaging manifestations of different fracture types, such as fracture displacement, and damage to accessory ligaments, tendons, and meniscus, type of joint cavity effusion, and damage to surrounding accessory bones were identified.

**Results:**

There were 21 tangential fractures (40.4%), 8 transverse fractures (15.4%), 8 longitudinal fractures (15.4%), 4 oblique fractures (7.7%), and 11 comminuted fractures (21.2%) among the 52 patients with patellar fracture. Tangential fractures begin at a younger age than the other four forms of fractures. When tangential fractures were compared to other types of fractures, medial patellar retinaculum and anterior and posterior cruciate ligament injuries were statistically significant (P< 0.05). The detection rate of trochlear dysplasia, type II and type III patellar was higher in patients with tangential fractures than in other fractures.

**Conclusions:**

Tangential fractures are less common to cause anterior and posterior cruciate ligament injuries than other types of fractures, but they are more likely to cause medial patellar retinaculum injuries.

## Introduction

The patellar, traversed by the quadriceps tendon, is the largest seed bone in the body and acts as a lever in the extensor apparatus of the knee, which is an important support structure for lower limb mobility [1]. Patellar fractures are rare relative to systemic fractures, accounting for less than 1% of systemic fractures [2]. There are few reports on patellar fractures, and most of the imaging technology focuses on X-rays or CT [3, 4], with few reports on MRI.

Patellar fractures can be caused by direct and (or) indirect injuries [4, 5]. Fractures were typified based on patellar fracture morphology, bone fragment displacement, and the number of fragments in research by Gwinner C [6] and Jarraya M [7]. While X-ray and CT scans were mostly used in prior investigations, MRI was the primary method of observation for the typing of patellar fractures in this study. MRI allows for not only the typing of patellar fractures but also the non-invasive evaluation of damage to surrounding accessory soft tissue structures, facilitating the understanding of the pathophysiological mechanisms of patellar fractures. To better understand the pathophysiological mechanisms of patellar fractures, MRI was utilized to identify the imaging signs of various types of patellar fractures.

## Materials and methods

### Patient cohort

The authors retrospectively collected clinical data and MRI images of 52 patients with patellar fractures from January 2019 to June 2022 at the Second Affiliated Hospital of Kunming Medical University. Inclusion criteria: (1) patients aged 18 years or older with patellar fracture; (2) complete clinical data and X-ray or CT scans. Exclusion criteria: (1) MR images of poor quality; (2) patients with tumors, inflammation, or infection in the knee region. The study population consisted of 19 men and 33 women. The age range was from 18 to 70 years, the median age was 37 years. This study was initiated in September 2022 with Institutional Review Board approval (No. PJ-2022-144) and informed consent was waived. All methods were carried out in accordance with relevant guidelines and regulations.

### Image acquisition

All patients underwent MRI examinations using a 1.5T superconducting MRI scanner (Alltech) fitted with flexible coils surface-based on the knee. Images were obtained in a supine position by extension of the patient’s knee and inward rotation of the toes 0~ 15°. Scanning parameters: T1-weighted spin-echo sequence and fat-suppressed proton density-weighted fast spin-echo sequence were performed in the transverse, sagittal, and coronal planes of the knee, respectively. The number of signal averages is 3 for the T1-weighted image and 7 for the fat-suppressed proton density image. The field of view was 180 mm, the slice thickness was 4mm, and the matrix was 256×256 for all studies.

### Image discrimination criteria

**Fig 1:**
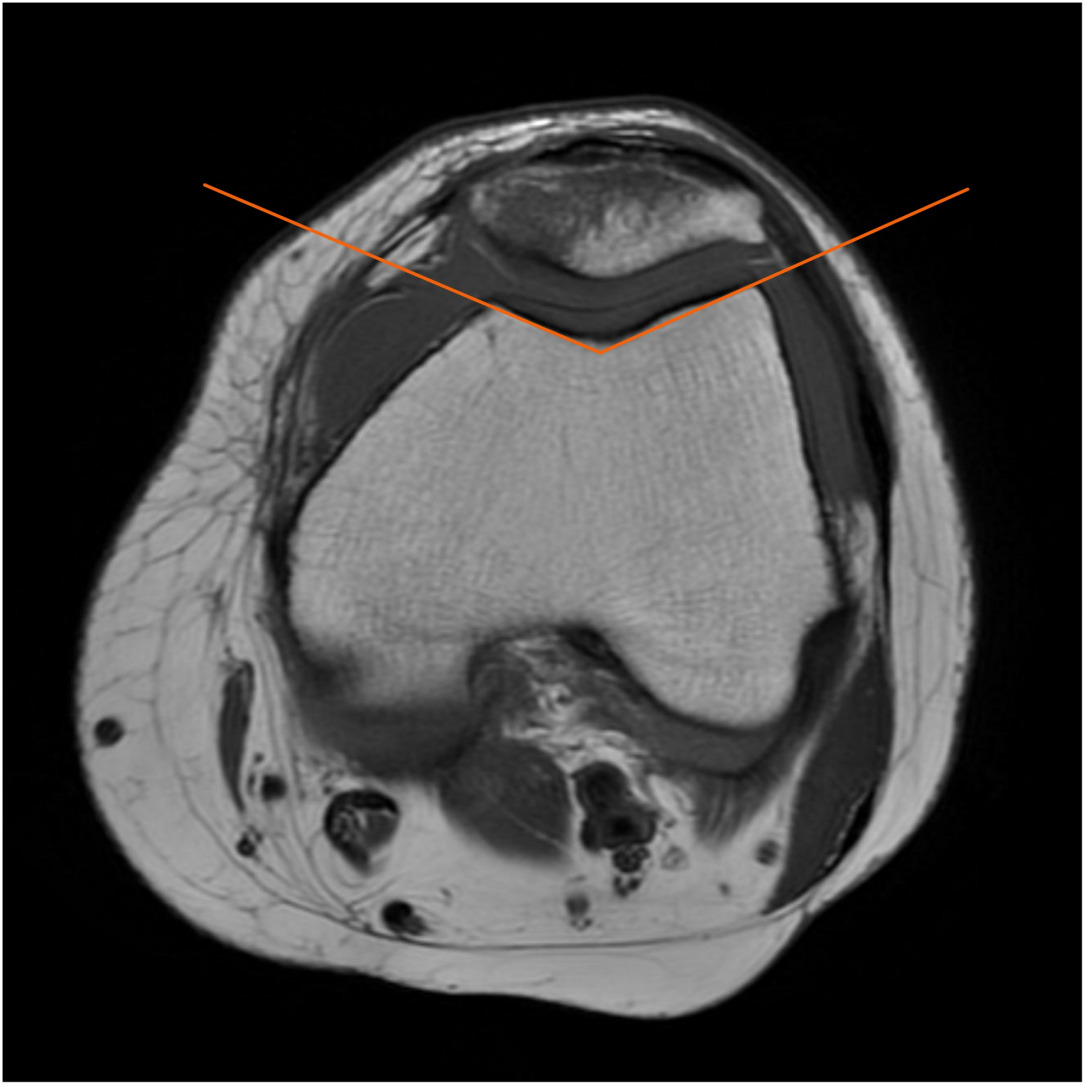
Measurement of trochlear groove angle on axial T1WI image.

For each case included in the study, the MRI image was independently examined by two radiologists (comparable years of work and more than 5 years of experience). Disagreements were resolved by consensus. The image discrimination criteria for the present study were as follows:(1) There are 5 types of patellar fractures. A: Transverse fracture. B: Longitudinal fracture. C: Oblique fracture. D: Tangential fracture. E: Comminuted fracture. (2) Fracture displacement. The fracture fragments are separated, and the distance between them is greater than 3 mm was determined to be a displaced fracture. (3) Ligament injuries include 3 grades (including the anterior and posterior cruciate ligament, the medial and lateral retinaculum, and the medial-lateral collateral ligament). Grade I: Ligament contusion. Ligaments are continuous and intact with blurred margins, accompanied by surrounding soft tissue edema and exudation. Grade II: Partial tear of the ligament. The ligaments are thickened, edematous, and oozing, with some direct signs of local rupture and surrounding soft tissue edema. Grade III: Complete rupture of the ligament fibers, in and around the fracture area can see edema and hemorrhage. (4) Quadriceps tendon and patellar tendon injury: a linear-like T2WI high signal (normally low signal) within the quadriceps tendon and patellar tendon, the tendon can be uninterrupted or ruptured. (5) Meniscal injury manifests as a linear-like high signal within the meniscus, which may extend to the capsule rim of the meniscus or the high signal within the meniscus up to one or both articular surfaces. (6) The capsular effusion was assessed with three different grades: Grade I: No effusion. Grade II: Simple effusion. Grade III: Complex effusion, including hemorrhagic effusion and hemolipid effusion. (7) Peripheral bone structural damage: Bone contusion or fracture of the femur, tibia, or fibula. (8) Trochlea dysplasia: Trochlea dysplasia is defined by a trochlear groove angle of more than 145 degrees (Fig 1). (9) Patellar morphology is divided into three types according to the Wiberg classification (Table 1) [8].

**Table 1:** Patellofemoral morphology Wiberg classification criteria.

Wiberg Classification	Classification Criteria
Wiberg type I	The ridge was seen to be situated approximately in the center of the patellar.
Wiberg type II	The ridge was situated slightly toward the medial border of the patellar, and the medial facet was smaller than the lateral.
Wiberg type III	The ridge was displaced medially to such a degree that there was hardly any room left over for the medial facet.

### Statistical analysis

All statistical analyses were performed with SPSS (version 26.0). Comparison of damage to various ligaments, tendons, and surrounding bones of the knee joint in different types of patellar fractures, with measured data expressed as ($\bar{x}\pm s$) and numerical data expressed as ratios or proportions of composition. The chi-square test or Fisher’s exact test was applied to compare the damage to various knee joint ligaments in different types of patellar fractures. Results with P values less than 0.05 were considered to be significant.

## Results

This study included 52 patella fractures. 31 right and 21 left patellar fractures. 18 were treated with surgery and 34 without operation.

### Patellar fracture types

**Fig 2:**
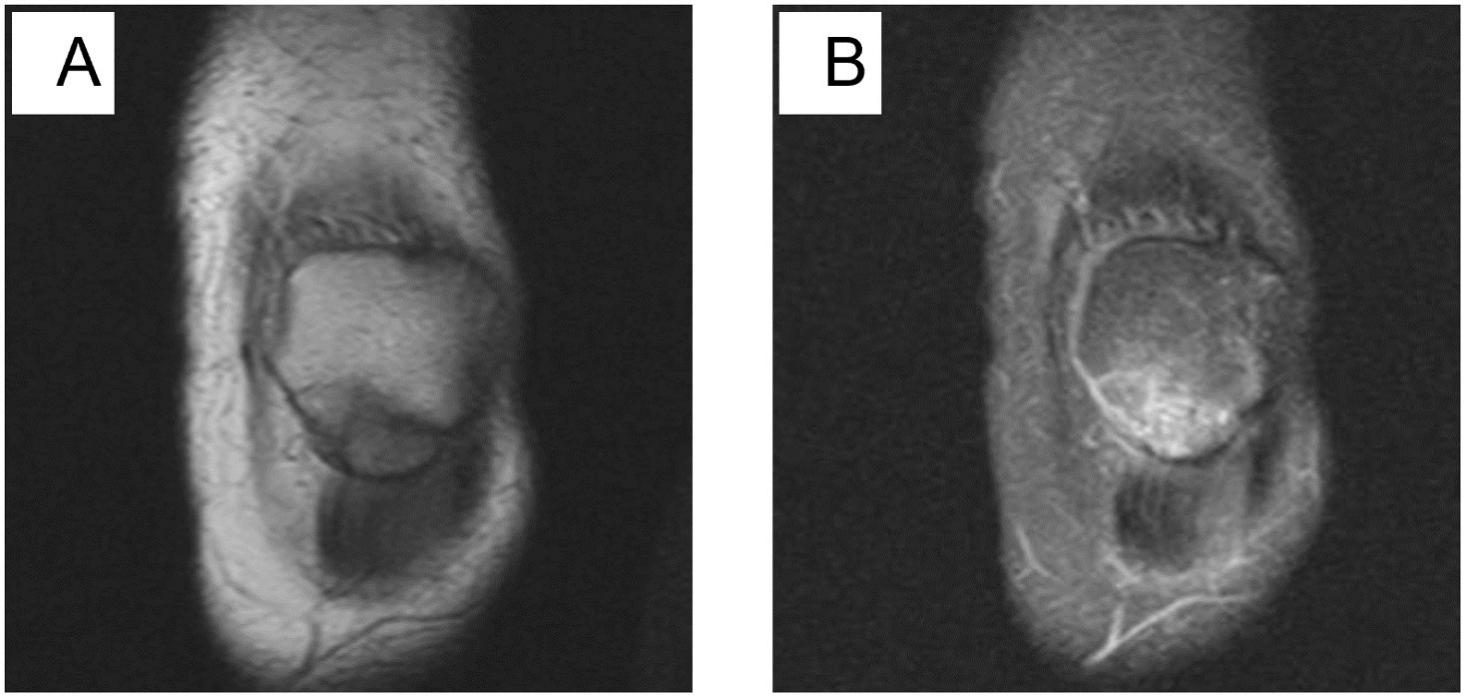
A: Coronal T1-weighted MR image; B: Coronal fat-suppressed proton density-weighted MR image. Female, 32 years old, transverse fracture. The patellar was divided into two parts: upper and lower.

**Fig 3:**
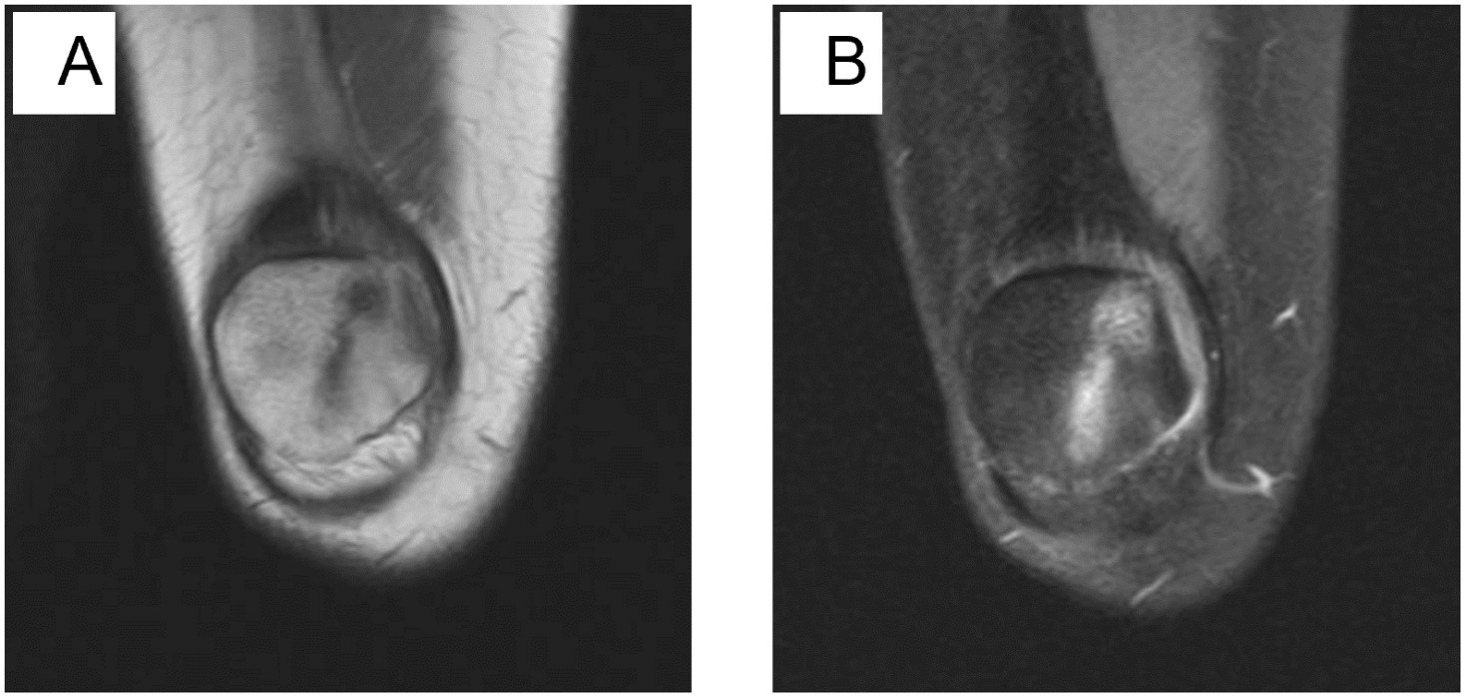
A: Coronal T1-weighted MR image; B: Coronal fat-suppressed proton density-weighted MR image. Female, 46 years old, longitudinal fracture. The T1WI signal of the patellar is reduced, and the patellar is divided into two parts: the right and the left.

**Fig 4:**
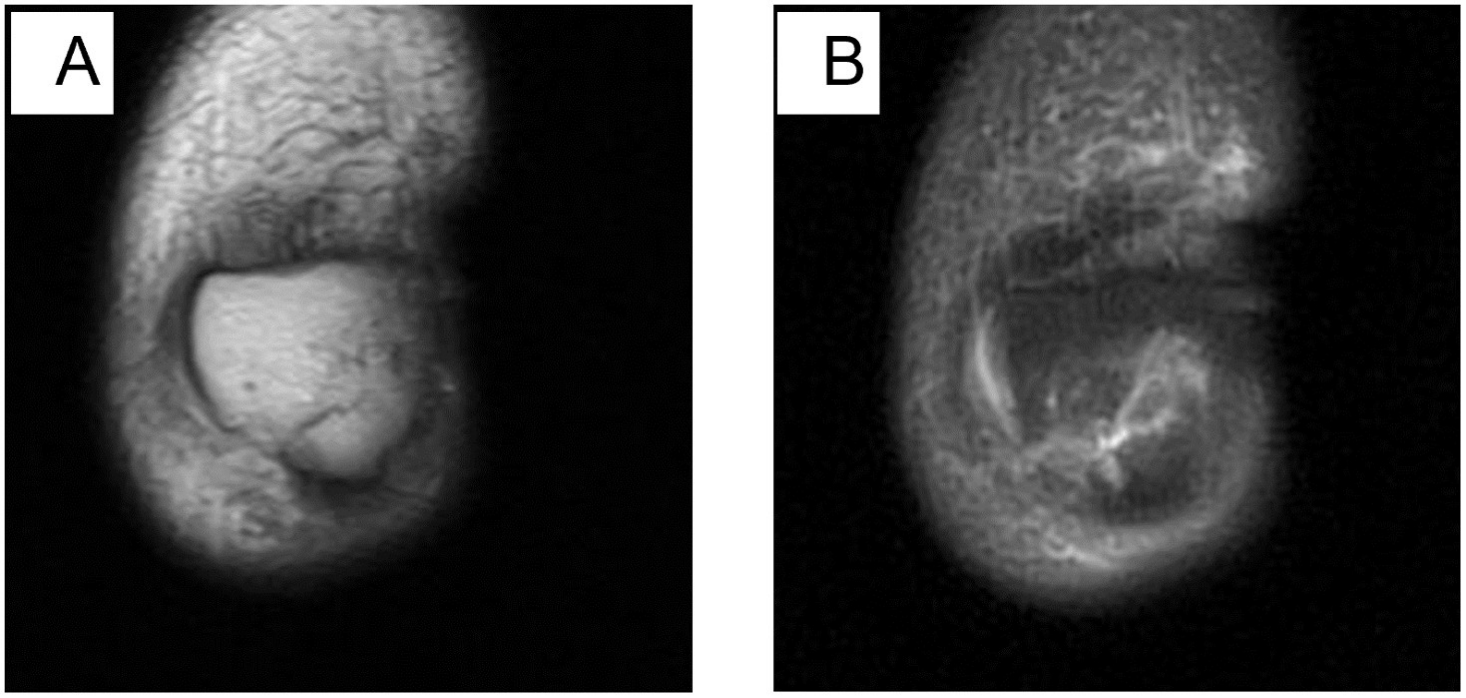
A: Coronal T1-weighted MR image; B: Coronal fat-suppressed proton density-weighted MR image. Male, 60 years old, oblique fracture. The fracture line is at an angle to the mid-axis of the body.

**Fig 5:**
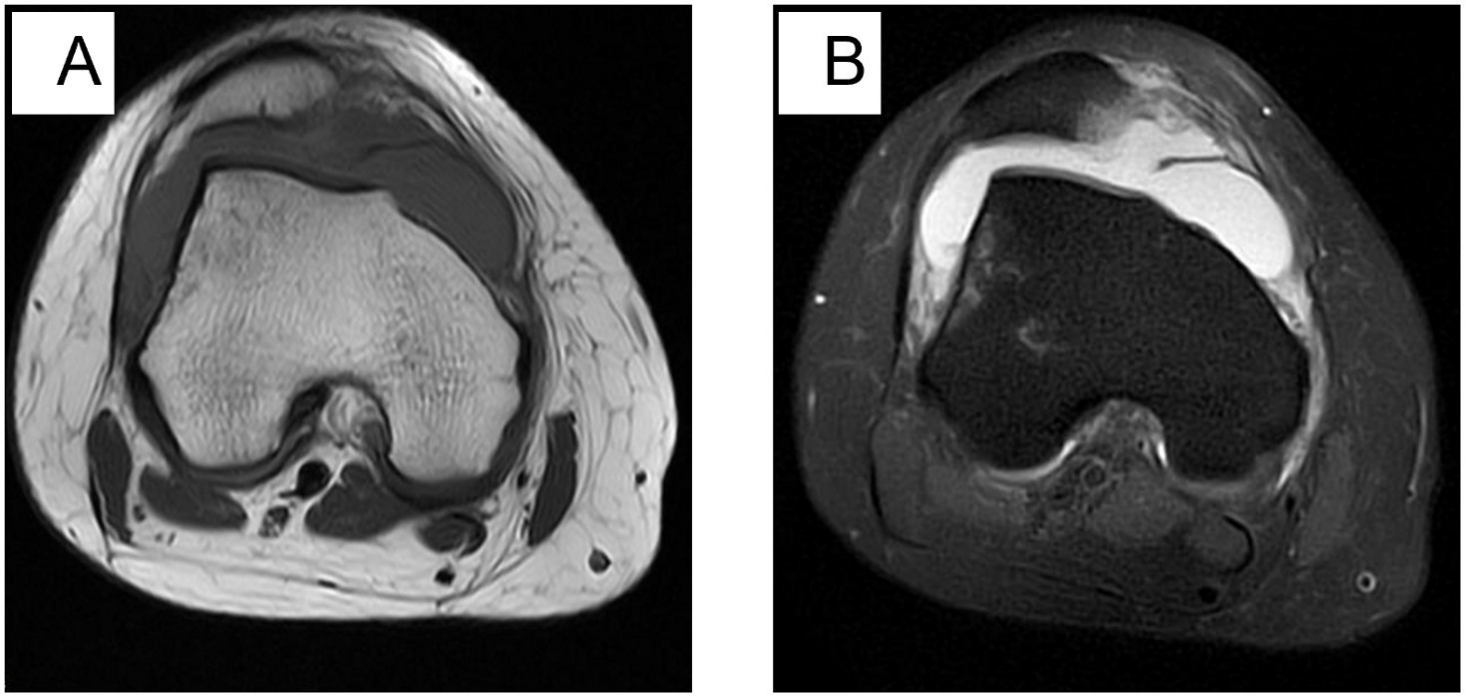
A: Axial T1-weighted MR image; B: Axial fat-suppressed proton density-weighted MR image. Female, 26 years old, tangential fracture. Patellar subluxation with localized osteochondral defects in the medial portion of the patellar, patchy osteoedema of the lateral femoral condyle on axial fat-suppressed proton density-weighted MR image, and grade II signal in the medial patellar retinaculum. Simple effusion in the joint capsule.

**Fig 6:**
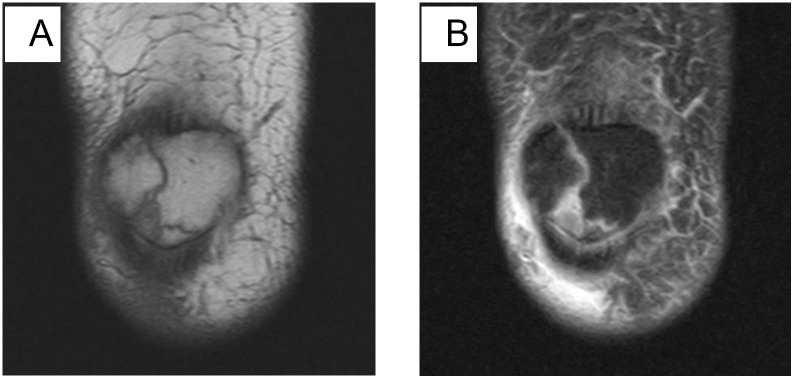
A: Coronal T1-weighted MR image; B: Coronal fat-suppressed proton density-weighted MR image. Male, 46 years old, comminuted fracture. The patellar was divided into 3 parts of unequal size and the subcutaneous soft tissue around the joint was edematous.

Among the 52 patients, there were 8 transverse fractures (15.4%)(Fig 2A and 2B), 8 longitudinal fractures (15.4%)(Fig 3A and 3B), 4 oblique fractures (7.7%)(Fig 4A and 4B), 21 tangential fractures (40.4%)(Fig 5A and 5B), and 11 comminuted fractures (21.2%)(Fig 6A and 6B).

The displaced fracture occurred in 28 of all fracture patients (53.8%), including 4 transverse fractures, 3 longitudinal fractures, 16 tangential fractures, and 5 comminuted fractures (Table 2).

**Table 2:** Sex and age of different types of patella fractures (n, %).

		Transverse fracture (n = 8)	Longitudinal fracture (n = 8)	Oblique fracture (n = 4)	Tangential fracture (n = 21)	Comminuted fracture (n = 11)
Sex (n, %)						
	Man	4 (50%)	2 (25%)	1 (25%)	6 (28.6%)	6 (54.5%)
	Woman	4 (50%)	6 (75%)	3 (75%)	15 (71.4%)	5 (45.5%)
Age		45.38±15.29	50.63±16.06	59.25± 3.59	23.29± 10.10	40.09± 14.75

The incidence of transverse and comminuted fractures was similar in men and women; for the other three fracture types, it is higher in women than in men. The age of onset for tangential fractures is lower than for the other four fracture types.

### Peripatellar tissue injury

#### Ligament injury

Among the 52 cases of patellar fractures combined with ligament injuries by MRI, there were 3 cases without any ligament injury (5.8%), including 1 case of transverse fracture, 1 case of longitudinal fracture, 1 case of tangential fracture; 23 cases of single ligament injury (44.2%), including 3 cases of transverse fracture, 2 cases of longitudinal fracture, 1 case of oblique fracture, 11 cases of tangential fracture, and 6 cases of comminuted fracture; 26 cases of multi-ligament injuries (50.0%), including 4 cases of transverse fractures, 5 cases of longitudinal fractures, 3 cases of oblique fractures, 9 cases of tangential fractures, and 5 cases of comminuted fractures, details of which are shown in Table 3. In the comparison of ligament injuries in the knee joint among different patellar fracture types, the injuries of the anterior (P = 0.002) and posterior cruciate ligaments (P = 0.007) and the medial patellar retinaculum (P = 0.001) were considered to be significant.

**Table 3:** Different types of patellar fractures combined with ligament injuries (n, %).

Fracture Type	n	Anterior Cruciate Ligament Injury	Posterior cruciate ligament injury	Medial patellar retinaculum injury	Lateral patellar retinaculum injury	Medial collateral ligament injury	Lateral collateral ligament injury
Transverse fracture	8	4(50.0%)	2(25.0%)	2(25.0%)	3(37.5%)	2(25.0%)	2(25.0%)
Longitudinal fracture	8	6(75.0%)	3(37.5%)	2(25.0%)	2(25.0%)	2(25.0%)	0(0.0%)
Oblique fracture	4	1(25.0%)	2(50.0%)	2(50.0%)	1(25.0%)	0(0.0%)	1(25.0%)
Tangential fracture	21	2(9.5%)	0(0.0%)	17(81.0%)	5(23.8%)	6(28.6%)	2(9.5%)
Comminuted fracture	11	5(45.5%)	2(18.2%)	5(45.5%)	3(27.3%)	1(9.1%)	2(18.2%)

#### Quadriceps tendon and patellar tendon injury

No quadriceps tendon injury was discovered in tangential fractures, and all patellar fractures were combined with a patellar tendon injury. As shown in Table 4, among them, transverse and oblique fractures had a higher rate of patellar tendon injury than other fracture types.

**Table 4:** Peripatellar tissue injuries in patients with different types of patellar fracture (n, %).

Peripatellar tissue injury		Transverse fracture (n = 8)	Longitudinal fracture (n = 8)	Oblique fracture (n = 4)	Tangential fracture (n = 21)	Comminuted fracture (n = 11)
Tendon Injury	Quadriceps tendon injury	1(12.5%)	2(25.0%)	1(25.0%)	0(0.0%)	4(36.4%)
	patellar tendon injury	7(87.5%)	4(50.0%)	3(75.0%)	2(9.5%)	7(63.6%)
Meniscus injury	Medial meniscus injury	2(25.0%)	0(0.0%)	0(0.0%)	0(0.0%)	3(27.3%)
	Lateral meniscus injury	2(25.0%)	1(12.5%)	1(25.0%)	4(19.0%)	1(9.1%)
Joint capsule effusion	No effusion	0(0.0%)	0(0.0%)	0(0.0%)	1(4.8%)	0(0.0%)
	Simple effusion	3(37.5%)	8(100.0%)	4(100.0%)	14(66.7%)	3(27.3%)
	Complex effusion	5(62.5%)	0(0.0%)	0(0.0%)	6(28.6%)	8(72.7%)

#### Meniscal injury

Only longitudinal, oblique, and tangential fractures combined with lateral meniscal injury, whereas both medial and lateral meniscal injuries were seen in transverse and comminuted fractures, as detailed in Table 3.

#### Joint capsule effusion

One patient with a tangential patellar fracture had no effusion in the joint capsule, while the other 35 patients with patellar fractures had effusion in the joint capsule. Simple exudates were present in all fracture types, with a higher percentage of transverse and comminuted fractures combined with complex exudates, as detailed in Table 4.

#### Peripheral bone structural damage

One of the transverse fractures (8 cases) was combined with a fracture of the lower femur, two with a bone contusion of the lower femur, and two with a bone contusion of the tibial plateau; a longitudinal fracture (8 cases) was accompanied by a fracture of the lateral femoral condyle, and one case was associated with a bone contusion under the tibial plateau and the fibula head; one of the oblique fractures (4 cases) was combined with an internal posterior fracture of the distal femur; bone contusions of the lateral femoral condyle occurred in 19 of the tangential fractures (21 cases) and 2 cases combined with bone contusions of the tibial plateau; one case of comminuted fracture (11 cases) was accompanied by tibial plateau fracture and medial femoral condyle bone contusion, and two cases were accompanied by distal femoral medial-posterior fracture and proximal tibial bone edema.

#### Trochlear and patellar morphology

In tangential fractures, only one case had well-developed trochlea, with a higher likelihood of trochlear dysplasia compared to other types of fractures, and there was a statistically significant difference. In tangential fractures, the patellar morphology is predominantly type II and type III. And the detection rate of type III patellar is higher than that of other types of fractures, while the detection rate of type I patellar is lower than that of other fractures, as detailed in Table 5.

**Table 5:** Comparison of patellofemoral joint morphology among patients with different types of patellar fractures (cases, %).

Fracture type	Cases	Trochlear dysplasia	Patellar morphology type		
			type I	type II	type III
Tangential fracture	21	20(95.2%)	3(14.3%)	10(47.6%)	8(38.1%)
Other types of fractures	31	20(64.5%)	17(54.8%)	13(41.9%)	1(3.2%)
P value	-	0.025^※^	0.003^※^	0.686	0.002^※^

^※^ means p< 0.05, the difference is statistically significant.

## Discussion

### The value of MRI in typing patellar fractures

The more popular classification AO (Arbeitsgemeinschaft für Osteosynthesefragen), is based on the number of fracture fragments, whether or not they are displaced, and whether cartilage is involved by viewing radiographic and CT images [6]. In this study, the classification was based solely on fracture morphology. This study was classified patellar fractures into 5 types: transverse fractures, longitudinal fractures, oblique bones, tangential fractures, and comminuted fractures, of which tangential fractures were the most common (40.4%), this was different from that reported by Meenen [9] et al. (The patellar fracture was divided into 6 types: type A, simple transverse fracture; type B, simple oblique fracture; type C, avulsion fracture; type D, simple longitudinal fracture; type E, simple comminuted fracture; and type F, complex comminuted fracture. Type A patellar fracture was the most common) [10]. The apparent difference between the two studies is due to the following two factors: (1) Meenen et al. did not include patients with tangential patellar fractures in their study. Tangential fractures are more common than other types of patellar fractures and often occur in patients with patellofemoral joint instability. Patients with patellofemoral joint instability have a larger base and therefore a higher incidence of patellofemoral joint instability. (2) Meenen et al. primarily determined the presence of patellar fractures and the type of fracture based on X-ray or CT scan results [11–14]. However, it is difficult for X-rays or CT scans to detect the presence of tangential patellar fractures: A: Patellar tangential fractures occur mostly in the patellar crest or just below the medial articular surface of the patellar. When X-rays are taken, this area is obscured by anatomical structures just anterior and posterior to it, which can lead to missed lesions. B: Tangential patellar fractures have small fragments and are often easily missed if not carefully examined on CT. C: In some cases of tangential patellar fracture, the fracture line involves only the cartilage of the patellar and does not involve the bone below the patellar cartilage. Therefore, it is also difficult to detect the presence of tangential patellar fracture on conventional X and CT examination. And this study used MRI to visualize the lesions. MRI has the advantage of multidirectional and multiparametric imaging [15] to show the location and morphology of the patellar fracture.

### Mechanisms of different patellar fractures and corresponding imaging changes

Transverse fractures are usually the result of two forces acting on the patellar, one directed upward (active forces) and the other downward (passive forces) [16]. The quadriceps quickly contracts and produces a significant tensile force during rapid knee flexion. When this stress exceeds the force that the patellar can withstand, a transverse fracture occurs (the fragment splits into two parts, the upper and the lower) [17], and there may even be a fragment cephalad displacement of the fragment [18].

In this study, three (37.5%) transverse fractures were associated with damage to the lateral patellar retinaculum, and two (25.0%) were associated with damage to the medial patellar retinaculum. This may be due to the quadriceps muscle contracts strongly during fracture and radiates its force to both sides of the patellar, resulting in damage to the medial and lateral retinaculum [16]. In addition, four cases (50.0%) of this type had an anterior cruciate ligament injury, the cause of which is unknown and may be related to the anterior position of the anterior cruciate ligament and its susceptibility to external forces. Meanwhile, three cases (37.5%) of transverse fractures were accompanied by meniscus injury, which may be related to the quadriceps muscle contracts strongly that caused internal or external rotation of the femur during the fracture [19].

When knee bend, the patellar moves outward (a portion of the patellar extends beyond the anterolateral border of the lateral femoral condyle). At this point, the anterolateral edge of the lateral femoral condyle acts as a "wedge", dividing the patellar longitudinally into left and right parts under the direct influence of anterior forces. A portion of the patellar (the portion located laterally) extends beyond the anterolateral edge of the lateral femoral condyle, and therefore longitudinal fractures tend to occur at the lateral edge of the patellar. That is why longitudinal fractures are also called marginal fractures [2]. In this type of patellar fracture, the fracture fragments may be displaced by the action of the medial or lateral patellar retinaculums (inward and outward tensile forces). In this type of patellar fracture, there were 3 cases with fracture displacement, accounting for 37.5%. In addition, the tendon along the fracture line is also vulnerable to injury [20]. In this type of patellar fracture, there were 4 cases with a patellar tendon injury, accounting for 50.0%. Moreover, of the patients who had a longitudinal fracture of the patellar, 4 had an injury to the patellar retinaculum (accounting for 50.0%). This can be caused by rapid stretching of the patellar retinaculum during rapid knee flexion, resulting in patellar retinaculum injury [21]. Meanwhile, of the patients who had longitudinal fractures, six (75.0%) had anterior cruciate ligament injuries and three (37.5%) had posterior cruciate ligament injuries. This may be due to the fact that, the femur undergoes torsion when force is applied to the lateral femoral condyle, which can lead to cruciate ligament injuries [22].

The oblique fractures are usually the result of a combination of factors that cause both transverse and longitudinal fractures. That is, the oblique fracture occurred with both a strong upward contractile force of the quadriceps femoris muscle and an external force acting directly on the patellar. Of our cases of oblique fractures, 3 (75.0%) had patellar tendon injuries. This should be related to the pathogenesis of the disease (both external forces and ligamentous tension act directly on the patellar tendon anterior to the patellar) [23]. In addition of the oblique fractures, 2 cases (50.0%) had the medial patellar retinaculum injuries and the other case (25.0%) had the lateral patellar retinaculum injury. This should be the same principle as a transverse fracture causing injury to the medial and lateral patellar retinaculums.

No fracture displacement was found in the oblique fracture group, which may be related to the fact that oblique fractures are the result of the interaction of different types of forces (There is no apparent dominant force, and therefore the fracture fragments in the above directions are less likely to be displaced). This situation may also be related to the small number of oblique fracture cases collected in this study.

In addition, the joint capsule effusions in all four cases of oblique patellar fractures were simple effusions. This may be related to the fact that the fracture fragments are not significantly displaced or that the fracture line does not penetrate the patellofemoral articular surface of the patellar (the fracture line does not communicate with the articular bursa) [24].

Tangential fractures are often due to anatomical factors such as patellofemoral dysplasia and long retinacular ligaments [25]. Tangential fractures usually occur when the patient is in a standing position with a semi-flexed knee (after knee valgus or torsion). The patellar moves outward under an outward force. At the same time the patellar crest of the patellar collides with the lateral femoral condyle. Subsequently, the patellar, in the process of repositioning, the medial articular surface of the patellar or the inferior portion of the patellar crest again collides with the lateral femoral condyle [26]. Both collisions are point (the medial patellar articular surface or the lower part of the patellar crest) and facet (lateral femoral condyle) impacts. Therefore, it is prone to fracture of the medial patellar, as well as detachment and displacement of the patellar cartilage and osteochondral bone of the patellar [27]. In this type, only one case had a well-developed trochlea, while the rest exhibited trochlear dysplasia. And the incidence of type II and III patellar was higher than other types of fractures. The study by Cao et al. shows that the closer the patellar morphology is to Wiberg III type, the weaker the stability of the patellofemoral joint [8]. This suggests that patients with tangential position fractures often have patellofemoral instability, increasing the risk of outward patellar dislocation. Otherwise, 16 (76.2%) of the tangential fractures were combined with cartilage or bone displacement, and the incidence was significantly higher than other fracture types. This could be because MRI is more sensitive to cartilage or osteochondral defects, as it clearly visualizes the cartilage and bony bone of the patellar and the process of two collisions increases the chance of cartilage or osteochondral damage and displacement.

In addition, when the patellar was dislocated outward, the medial patellar retinaculum was easily stretched and injured [28]. Among the 21 tangential fractures in this group, 17 cases (81.0%) were combined with injury to the medial patellar retinaculum, and the incidence was significantly higher than other types of patellar fractures (P < 0.05).

The majority of comminuted fractures are caused by an external force acting directly on the patella (such as a fall or impact), which exerts a powerful force on the knee’s anterior portion [29], and exposes the surrounding bone and supporting structure to injury [16]. One of the 11 cases of this type was accompanied by a fracture of the tibial plateau and bone contusion of the medial femoral condyle, and two other cases were accompanied by a distal femoral medial-posterior fracture and bone edema of the proximal tibial segment. Furthermore, this type of patellar fracture is often combined with damage to the ligaments (including the cruciate ligament, the patellar retinaculum and lateral collateral ligament), tendons, and the meniscus. This may be due to the force acting directly on the patellar is so strong that the knee joint twists in different directions because the force is transmitted in all directions [22], resulting in damage to the support apparatus.

Of these 11 cases of comminuted patellar fracture, eight (72.7%) were combined with complex capsular effusion of the joint capsule (hemorrhagic effusion or hemolipid effusion), which is similar to the incidence of transverse fractures with complex capsular effusion of the joint capsule (5 cases, 62.5%). This could be due to these two types of patellar fractures are subjected to greater stresses, with more damage to the adjacent bone and soft tissues, and are more prone to vascular rupture and lipid droplet penetration from the cancellous bone into the joint capsule [24].

There are advantages to using MRI for fracture diagnosis in this study, there are limitations which included: Small population, more patients should be followed up on in future research. Several cases lacked confirmation of surgical intervention, and future studies will enroll more surgically treated patients for follow-up. Although this study has limited clinical implications for treatment, it is of great help in understanding the pathophysiology of patellar fractures.

## Conclusion

The study allows the typology of patellar fractures by adding tangential fracture as a fracture type. The multi-directional and multi-parametric imaging capabilities of an MRI examination can clearly display the location and morphology of a patellar fracture as well as the soft tissue damage that surrounds it.
